# The effect of a diet based on rice straw co-fermented with probiotics and enzymes versus a fresh corn Stover-based diet on the rumen bacterial community and metabolites of beef cattle

**DOI:** 10.1038/s41598-020-67716-w

**Published:** 2020-07-01

**Authors:** Yongqiang Hu, Yuyong He, Shan Gao, Zhiqiang Liao, Tao Lai, Haimei Zhou, Qianlin Chen, Lingyu Li, Haijun Gao, Wei Lu

**Affiliations:** 1 Jiangxi Province Key Laboratory of Animal Nutrition/Engineering Research Center of Feed Development, Jiangxi Agricultural University, Nanchang, China; 2 Yichun Institute of Agriculture Science in Jiangxi Province, Yichun, China; 3 Yichun Institute of Agricultural Varieties, Yichun, China

**Keywords:** Animal physiology, Bacteria

## Abstract

Improvement of the food value of rice straw is urgently required in rice crop growing areas to mitigate pollution caused by rice straw burning and enhance the supply of high-quality forages for ruminants. The aims of the present study were to compare the effects of fresh corn Stover and rice straw co-fermented with probiotics and enzymes on rumen fermentation and establish the feasibility of increasing the rice straw content in ruminant diets and, by extension, reducing air pollution caused by burning rice straw. Twenty Simmental hybrid beef cattle were randomly allotted to two groups with ten cattle per group. They were fed diets based either on rice straw co-fermented with probiotics and enzymes or fresh corn Stover for 90 days. Rumen fluid was sampled with an esophageal tube vacuum pump device from each animal on the mornings of days 30, 60, and 90. Bacterial diversity was evaluated by sequencing the V4–V5 region of the 16S rRNA gene. Metabolomes were analyzed by gas chromatography/time-of-flight mass spectrometry (GC–TOF/MS). Compared to cattle fed fresh corn Stover, those fed rice straw co-fermented with probiotics and enzymes had higher (*P* < 0.05) levels of acetic acid and propionate in rumen liquid at d 60 and d 90 respectively, higher (*P* < 0.05) abundances of the phyla Bacteroidetes and Fibrobacteres and the genera *Ruminococcus*,* Saccharofermentans*,* Pseudobutyrivibrio*,* Treponema*, *Lachnoclostridium*, and *Ruminobacter*, and higher (*P* < 0.05) concentrations of metabolites involved in metabolisms of amino acid, carbohydrate, and cofactors and vitamins. Relative to fresh corn Stover, rice straw co-fermented with probiotics and enzymes resulted in higher VFA concentrations, numbers of complex carbohydrate-decomposing and H_2_-utilizing bacteria, and feed energy conversion efficiency in the rumen.

## Introduction

It is of great significance to vigorously develop ruminant production in the main grain producing areas to increase the amount of crop straw in ruminant diet, reduce the pollution caused by crop straw burning, and increase the income of farmers and herdsmen. However, feeding large amounts of untreated crop straw to ruminants will reduce their performance and expel large amounts of methane, which not only reduces the energy efficiency of ruminant diet, but also adversely affects the climate^1^. Therefore, the key problem to be solved is how to improve the effective fermentation of crop straw and reduce the formation of methane in the rumen while using crop straw to develop ruminant production.

During rumen fermentation, Firmicutes are the main H_2_ producing bacteria and Bacteroidetes are the net H_2_ utilizers^2^, a decrease in Firmicutes abundance and an increase in Bacteroidetes abundance can effectively reduce the production of methane^3^. Studies also reported that an increase of *Prevotella* genus can decrease methane production^3^, because *Prevotella* can increase propionate level and inhibit methanogenesis^4^. In addition, an increase in fumarate or some amino acids (aspartate, valine, leucine, isoleucine, glutamate) in rumen fluid benifits the production of propionate^3^. Low rumen pH favors propionate producing bacteria, but inhibits other microorganisms^5^. Starch and neutral detergent fiber (NDF) content and NDF digestibility of diet can affect the fermentation characteristics in rumen^6^, because dietary starch increases propionate production and decreases CH_4_^7^, and dietary NDF enhances the production of acetic acid and methane. Dietary fiber is a major energy source for ruminants in many parts of the world^8^. Thus, the efficient conversion of fiber in the rumen is vital to ruminant production. If ruminants could more efficiently utilize rice straw as a roughage source, then feed shortages and air pollution from rice straw burning and methane emission could be substantially mitigated.

Rice straw and fresh corn Stover are the common crop straws, which consist mainly of complex lignocellulose polymers, pectin, silica, and wax^9^. The cuticle-wax-silica layer and lignin hinder microbial and enzyme access to cellulose and hemicellulose^10,11^. Compared with corn Stover, rice straw has lower nutrient digestibility in the rumen as it has a thick outer cuticle-wax silica-layer and high lignin content^12,13^. For this reason, rice straw is usually burned instead of being used as the main constituent of ruminant diets.

Biological degradation is an increasingly popular alternative to crop straw pretreatment, because it decomposes cellulosic polymers to cellulose that can then be digested by various cellulases and hemicellulases^14,15^. Previous study by Wadhwa et al.^16^ showed that the intake and nutritive value of naturally fermented rice straw with urea were superior to those of untreated rice straw. However, there is little information on the effects of rice straw co-fermented with probiotics and enzymes on the rumen microbiome and metabolome. Fresh corn Stover is also used as ruminant roughage in China and it has higher feed values than untreated rice straw^17,18^. Here, we fermented rice straw with a mixture of *Bacillus subtilis*, *Enterococcus faecalis*, cellulase, and xylanase for 14 d. We then conducted an experiment to compare the influences of rice straw co-fermented with probiotics and enzymes with fresh corn Stover on beef cattle. Rumen fluid samples were collected to (1) compare rumen fermentation characteristics in cattle fed fermented rice straw with those fed fresh corn Stover; (2) investigate the differences in the compositions of H_2_-producing and utilizing bacteria and metabolites of the rumen fluid of cattle fed fermented rice straw with those fed fresh corn Stover; and (3) explore the relationship between the differential bacteria and differential metabolites in rumen fluid.

## Results

### Dry matter intake

During experiment, each day beef cattle had an average dry matter intake of (7.95 ± 0.08) kg in fermented rice straw group and (7.85 ± 0.10) kg in fresh corn Stover group, respectively, and no significant difference was observed between two groups (*P* = 0.433).

### Rumen pH, lactic acid, and VFA

Table 1 shows that beef cattle fed fermented rice straw based diet had lower pH value and lower lactic acid concentration in rumen fluid compared to beef cattle fed fresh corn Stover based diet at d 30 (*P* = 0.495 and *P* = 0.664), d 60 (*P* = 0.634 and *P* = 0.640) and d 90 (*P* = 0.257 and *P* = 0.834), respectively. Beef cattle fed fermented rice straw based diet had higher rumen concentrations of acetic acid and propionate than those fed fresh corn Stover based diet at d 60 (*P* = 0.022 and *P* = 0.023) and d 90 (*P* = 0.043 and *P* = 0.014), respectively.

**Table 1:** pH, lactic acid, and VFA in rumen fluid of cattle fed fermented rice straw- or fresh corn Stover-based diets.

Items	Sampling time	Fermented rice straw group (Group A)	Fresh corn Stover group (Group B)	*P*
pH	d 30 of feeding	6.12 ± 0.06	6.19 ± 0.07	0.495
	d 60 of feeding	6.18 ± 0.05	6.22 ± 0.05	0.634
	d 90 of feeding	6.31 ± 0.02	6.45 ± 0.11	0.257
Lactic acid (mM)	d 30 of feeding	0.30 ± 0.12	0.37 ± 0.08	0.664
	d 60 of feeding	0.65 ± 0.12	0.72 ± 0.09	0.640
	d 90 of feeding	0.44 ± 0.09	0.49 ± 0.20	0.834
Acetic acid (mM)	d 30 of feeding	38.12 ± 3.19	38.26 ± 3.30	0.976
	d 60 of feeding	49.29 ± 3.29	36.30 ± 3.22	0.022
	d 90 of feeding	48.22 ± 6.93	30.38 ± 2.63	0.043
Propionate (mM)	d 30 of feeding	9.00 ± 0.83	8.70 ± 0.90	0.809
	d 60 of feeding	16.61 ± 1.33	11.82 ± 1.07	0.023
	d 90 of feeding	10.39 ± 1.33	5.99 ± 0.47	0.014
Butyric acid (mM)	d 30 of feeding	7.93 ± 0.50	8.04 ± 0.81	0.907
	d 60 of feeding	10.34 ± 1.09	7.87 ± 0.82	0.108
	d 90 of feeding	4.80 ± 0.57	3.82 ± 0.55	0.255
Acetic:propionic	d 30 of feeding	4.26 ± 0.14	4.46 ± 0.24	0.499
	d 60 of feeding	2.98 ± 0.13	3.08 ± 0.12	0.596
	d 90 of feeding	4.62 ± 0.15	5.06 ± 0.17	0.092

### Bacterial community in rumen fluid

The sequence data produced in this experiment have been deposited in the National Center for Biotechnology Information (NCBI) Sequence Read Archive (SRA) under accession number SRP140749. Data in Table 2 showed that there was no significant differences in Chao 1 estimator and Shannon estimator of rumen fluid bacteria between cattle in fermented rice straw group and cattle in fresh corn Stover group at d 30 (*P* = 0.296 and *P* = 0.167), d 60 (*P* = 0.633 and *P* = 0.194) and d 90 (*P* = 0.460 and *P* = 0.869), respectively.

**Table 2:** Comparison of alpha diversity of bacteria in rumen fluid.

Items	Sampling time	Fermented rice straw group (Group A)	Fresh corn Stover group (Group B)	*P*
Chao 1	d 30 of feeding	1,101.80 ± 26.19	1,069.80 ± 11.50	0.296
	d 60 of feeding	1,095.80 ± 35.33	1,072.00 ± 32.44	0.633
	d 90 of feeding	1,139.80 ± 47.27	1,099.60 ± 21.14	0.460
Shannon	d 30 of feeding	5.07 ± 0.07	5.19 ± 0.04	0.167
	d 60 of feeding	4.92 ± 0.09	4.75 ± 0.09	0.194
	d 90 of feeding	5.13 ± 0.12	5.10 ± 0.07	0.869

**Figure 1:**
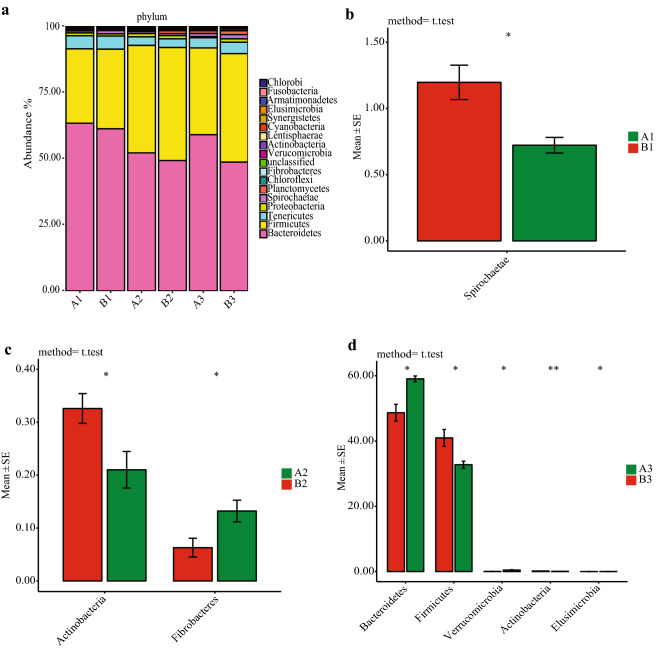
Changes of bacterial composition in fermented rice straw group and fresh corn Stover group. Bacterial composition at phylum level (**a**). The bacteria with significant difference in phylum level at d 30 (**b**), d 60 (**c**), d 90 (**d**) of feeding.

The composition and relative abundance of rumen fluid bacteria at the phylum level are depicted in Fig. 1a, Firmicutes and Bacteroidetes were the most dominant phyla (Table 3). Firmicutes and Bacteroidetes accounted for 28.12–40.63% and 52.15–63.37% of the total reads respectively in the rumen liquid of beef cattle fed fermented rice straw based diet, but for beef cattle fed fresh corn Stover based diet, Firmicutes and Bacteroidetes in rumen liquid represented 30.10–42.76% and 48.69–61.28% of the total reads respectively. Compared with the fresh corn Stover-fed cattle, the fermented rice straw-fed cattle had a lower relative abundance of Spirochaetae at d 30 (*P* = 0.018) (Fig. 1b), lower relative abundances of Actinobacteria (*P* = 0.033) and higher relative abundances of Fibrobacteres (*P* = 0.035) respectively at d 60 (Fig. 1c). At d 90, cattle fed fermented rice straw had lower relative abundances of Actinobacteria (*P* = 0.001) and Firmicutes (*P* = 0.030), higher relative abundances of Bacteroidetes (*P* = 0.013), Verrucomicrobia (*P* = 0.022) and Elusimicrobia (*P* = 0.041) than those fed fresh corn Stover respectively (Fig. 1d).

**Table 3:** The relative abundance of rumen fluid bacteria for the phyla level between two groups.

Items	A1	B1	A2	B2	A3	B3
Firmicutes	28.12 ± 0.76	30.10 ± 0.25	40.63 ± 0.92	42.76 ± 1.57	32.75 ± 0.48	40.97 ± 1.15
Bacteroidetes	63.37 ± 0.55	61.28 ± 0.29	52.15 ± 0.77	49.25 ± 1.82	59.07 ± 0.39	48.69 ± 1.15
Planctomycetes	0.49 ± 0.02	0.48 ± 0.05	0.55 ± 0.03	1.09 ± 0.15	1.02 ± 0.07	1.47 ± 0.10
Tenericutes	4.96 ± 0.15	4.94 ± 0.11	3.24 ± 0.08	3.27 ± 0.09	3.88 ± 0.25	4.33 ± 0.15
Proteobacteria	1.07 ± 0.13	0.83 ± 0.03	1.22 ± 0.05	1.18 ± 0.06	0.41 ± 0.04	1.30 ± 0.19
Spirochaetae	0.72 ± 0.03	1.20 ± 0.06	0.73 ± 0.03	0.77 ± 0.03	1.20 ± 0.02	1.57 ± 0.13
Lentisphaerae	0.15 ± 0.02	0.10 ± 0.01	0.23 ± 0.01	0.16 ± 0.03	0.08 ± 0.01	0.06 ± 0.01
Unclassified	0.12 ± 0.01	0.12 ± 0.02	0.20 ± 0.02	0.40 ± 0.03	0.18 ± 0.02	0.45 ± 0.04
Fibrobacteres	0.26 ± 0.02	0.35 ± 0.03	0.13 ± 0.01	0.06 ± 0.01	0.50 ± 0.07	0.20 ± 0.02
Chloroflexi	0.21 ± 0.02	0.27 ± 0.04	0.21 ± 0.02	0.43 ± 0.05	0.24 ± 0.04	0.61 ± 0.12
Verrucomicrobia	0.14 ± 0.01	0.11 ± 0.01	0.25 ± 0.07	0.12 ± 0.02	0.47 ± 0.05	0.07 ± 0.00
Actinobacteria	0.05 ± 0.00	0.06 ± 0.01	0.21 ± 0.02	0.33 ± 0.01	0.07 ± 0.01	0.18 ± 0.01
Synergistetes	0.05 ± 0.00	0.04 ± 0.00	0.05 ± 0.01	0.06 ± 0.01	0.03 ± 0.00	0.04 ± 0.01
Cyanobacteria	0.23 ± 0.03	0.05 ± 0.01	0.16 ± 0.02	0.08 ± 0.02	0.06 ± 0.01	0.04 ± 0.01
Armatimonadetes	0.02 ± 0.00	0.02 ± 0.00	0.02 ± 0.00	0.00 ± 0.00	0.00 ± 0.00	0.00 ± 0.00
Elusimicrobia	0.02 ± 0.00	0.04 ± 0.01	0.02 ± 0.00	0.02 ± 0.00	0.04 ± 0.00	0.02 ± 0.00
Fusobacteria	0.01 ± 0.00	0.01 ± 0.00	0.00 ± 0.00	0.02 ± 0.00	0.00 ± 0.00	0.00 ± 0.00
Chlorobi	0.01 ± 0.00	0.00 ± 0.00	0.00 ± 0.00	0.00 ± 0.00	0.00 ± 0.00	0.00 ± 0.00

A: rumen fluid sample collected from beef cattle fed fermented rice straw based diet. B: rumen fluid sample collected from beef cattle fed fresh corn Stover based diet. 1, 2, 3 means samples collected at d 30, d 60 and d 90, respectively. The data in the table are expressed as "Mean ± SE".

**Figure 2:**
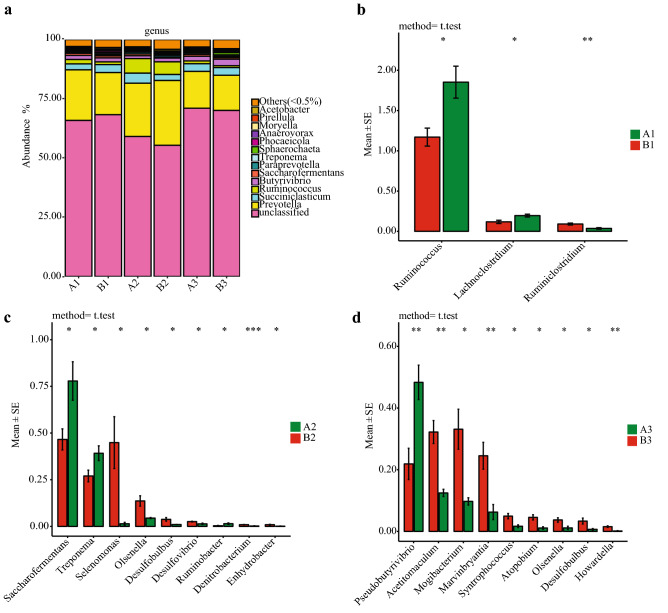
Changes of bacterial composition in fermented rice straw group and fresh corn Stover group. Bacterial composition at genus level (**a**). The bacteria with significant difference in genus level at d 30 (**b**), d 60 (**c**), d 90 (**d**) of feeding.

The composition and relative abundance of rumen fluid bacteria at the genus level are depicted in Fig. 2a. Rumen fluid bacteria with relative abundances > 0.5% and common to the cattle at various sampling times were: unclassified, *Prevotella, Succiniclasticum, Butyrivibrio, Ruminococcus,* and *Saccharofermentans*. Cattle fed fermented rice straw had higher relative abundances of *Ruminococcus* (*P* = 0.023) and *Lachnoclostridium* (*P* = 0.020), lower relative abundances of *Ruminiclostridium* (*P* = 0.007) at d 30 compared to those fed fresh corn Stover (Fig. 2b). At d 60, fermented rice straw-fed cattle had higher relative abundances of *Saccharofermentans* (*P* = 0.036)*, Treponema* (*P* = 0.043) and *Ruminobacter* (*P* = 0.043) respectively, lower relative abundances of *Selenomonas* (*P* = 0.035)*, Olsenella* (*P* = 0.030)*, Desulfobulbus* (*P* = 0.041)*, Desulfovibrio* (*P* = 0.035)*, Denitrobacterium* (*P* = 0.001) and *Enhydrobacter* (*P* = 0.010) than those fed fresh corn Stover respectively (Fig. 2c). At d 90, cattle fed fermented rice straw had higher relative abundances of *Pseudobutyrivibrio* (*P* = 0.008) and lower relative abundances of *Acetitomaculum* (*P* = 0.004)*, Mogibacterium* (*P* = 0.022)*, Marvinbryantia* (*P* = 0.009)*, Syntrophococcus* (*P* = 0.014)*, Atopobium* (*P* = 0.010)*, Olsenella* (*P* = 0.022)*, Desulfobulbus* (*P* = 0.048) and *Howardella* (*P* = 0.004) than those fed fresh corn Stover respectively (Fig. 2d).

**Figure 3:**
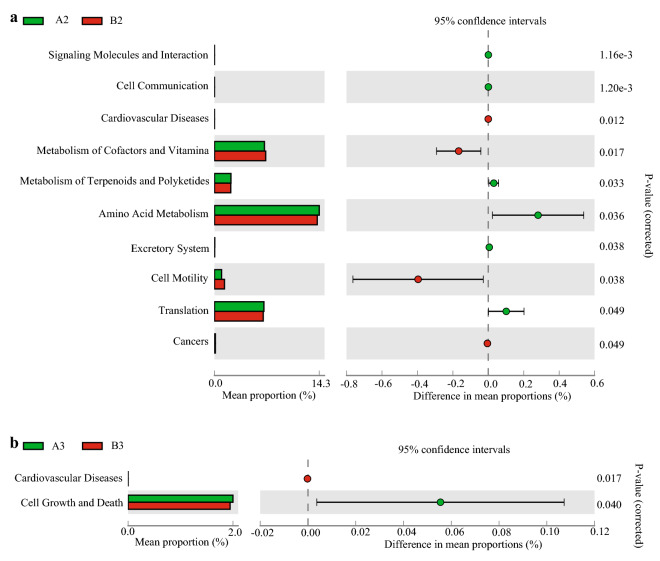
Bacteria function prediction in the rumen fluid of fermented rice straw group and fresh corn Stover group. The second level of KEGG pathway were showed in extended error bar at d 60 (**a**) and d 90 (**b**) of feeding.

**Figure 4:**
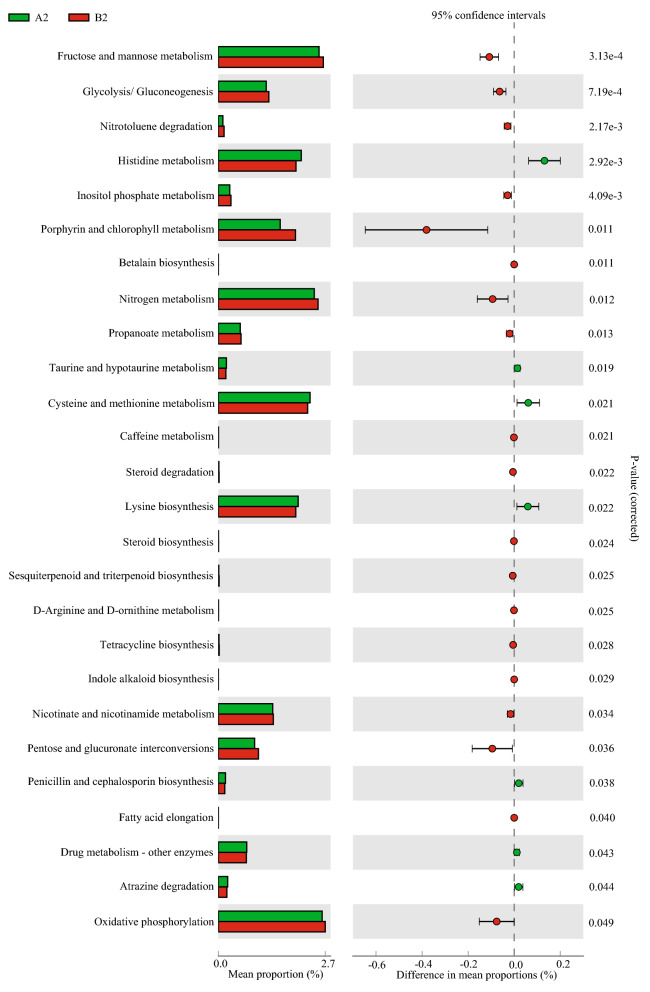
Bacteria function prediction in the rumen fluid of fermented rice straw group and fresh corn Stover group. The third level of KEGG pathway were showed in extended error bar at d 60 of feeding.

**Figure 5:**
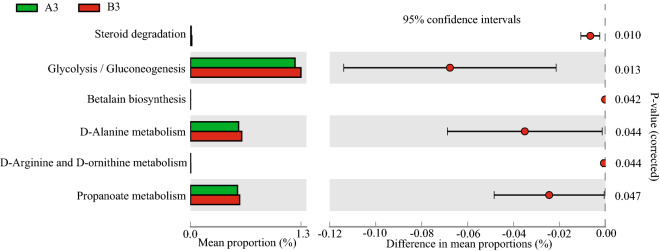
Bacteria function prediction in the rumen fluid of fermented rice straw group and fresh corn Stover group. The third level of KEGG pathway were showed in extended error bar at d 90 of feeding.

To assess relative differences between the fermented rice straw- and fresh corn Stover-fed cattle in terms of the predicted function of the microbiota in their rumen fluid, Tax4FUN was used to analyze their KEGG pathways. At the second KEGG level, no differences were detected between treatment groups in terms of the functions of their rumen fluid microbiota at d 30. At d 60, the rumen fluid of cattle fed fermented rice straw had higher relative abundances of microbiota involved in metabolism of terpenoid and polyketide (*P* = 0.033), amino acid metabolism (*P* = 0.036) and translation (*P* = 0.049) (Fig. 3a). At d 90, the rumen fluid of cattle fed fermented rice straw had higher relative abundances of microbiota involved in cell growth and death (*P* = 0.040) (Fig. 3b). At the third KEGG level, the rumen fluid of cattle fed fermented rice straw had higher relative abundances of microbiota involved in histidine metabolism (*P* = 0.002), cysteine and methionine metabolism (*P* = 0.021) and lysine biosynthesis (*P* = 0.022) at d 60 (Fig. 4). At d 90, the rumen fluid of cattle fed fermented rice straw had lower relative abundances of microbiota involved in glycolysis/gluconeogenesis (*P* = 0.013), *D*-alanine metabolism (*P* = 0.044) and propanoate metabolism (*P* = 0.047) (Fig. 5).

### Identified differential metabolites and metabolic pathways

The metabolites identified in the rumen fluid samples from cattle fed fermented rice straw and fresh corn Stover are listed in Table 4. Sixteen metabolites were identified including thirteen from rumen fluid sampled at d 30 and three from rumen fluid sampled at d 90. Fourteen compounds (benzoic acid, hydroxycinnamic acid, azelaic acid, phenylacetic acid, d-arabitol, fumaric acid, salicylic acid, adipic acid, 4-hydroxybenzoic acid, 2-methylglutaric acid, pimelic acid, 3-phosphoglycerate, xanthine, and adenosine) were upregulated and two compounds (threonine and glutamic acid) were downregulated in the rumen fluids of cattle fed fermented rice straw compared with those fed fresh corn Stover. The metabolic pathway enrichment analysis in Table 5 shows that the metabolites identified in the rumen fluid of the cattle fed fermented rice straw and fresh corn Stover participate primarily in amino acid metabolism followed by carbohydrate metabolism, cofactors and vitamins metabolism, nucleotide metabolism and biosynthesis of other secondary metabolites.

**Table.4:** Differential metabolites identified in rumen fluid.

Sampling time	Differential metabolites	Fermented rice straw group (Group A)	Fresh corn Stover group (Group B)	Fold change	*P*
d 30 of feeding	Benzoic acid	1.94 ± 0.37	0.70 ± 0.08	2.77	0.040
	Hydrocinnamic acid	100.45 ± 19.49	40.55 ± 8.90	2.48	0.037
	Azelaic acid	5.17 ± 1.07	1.82 ± 0.21	2.84	0.047
	Phenylacetic acid	17.66 ± 4.33	3.50 ± 0.83	5.05	0.042
	d-arabitol	0.53 ± 0.10	0.18 ± 0.02	2.94	0.035
	Fumaric acid	0.87 ± 0.17	0.34 ± 0.05	2.56	0.049
	Threonine	0.04 ± 0.02	0.19 ± 0.05	0.21	0.045
	Salicylic acid	0.19 ± 0.03	0.08 ± 0.01	2.38	0.029
	Adipic acid	0.31 ± 0.06	0.10 ± 0.02	3.10	0.034
	Glutamic	0.02 ± 0.02	0.18 ± 0.05	0.11	0.037
	Hydroxybenzoic acid	0.07 ± 0.01	0.02 ± 0.00	3.50	0.047
	Methylglutaric acid	0.03 ± 0.01	0.01 ± 0.00	3.00	0.039
	Pimelic acid	0.20 ± 0.04	0.06 ± 0.01	3.33	0.033
d 90 of feeding	Adenosine	0.16 ± 0.04	0.03 ± 0.02	5.33	0.037
	Xanthine	1.67 ± 0.40	0.45 ± 0.17	3.71	0.036
	Phosphoglycerate	0.04 ± 0.01	0.01 ± 0.01	4.00	0.017

**Table.5:** Enrichment analysis of KEGG pathway of differential metabolites.

Metabolism pathway	Differential metabolites
Amino acid metabolism	Benzoic acid, Phenylacetic acid, fumaric acid, hydrocinnamic acid, salicylic acid, 3-Hydroxybenzoic acid, glutamic, threonine
Carbohydrate metabolism	Fumaric acid, glutamic, d-arabitol
Cofactors and vitamins metabolism	Fumaric acid, glutamic, pimelic acid
Nucleotide metabolism	Adenosine, xanthine
Biosynthesis of other secondary metabolites	Xanthine

### Correlation analysis between differential bacteria and differential metabolites

**Figure 6:**
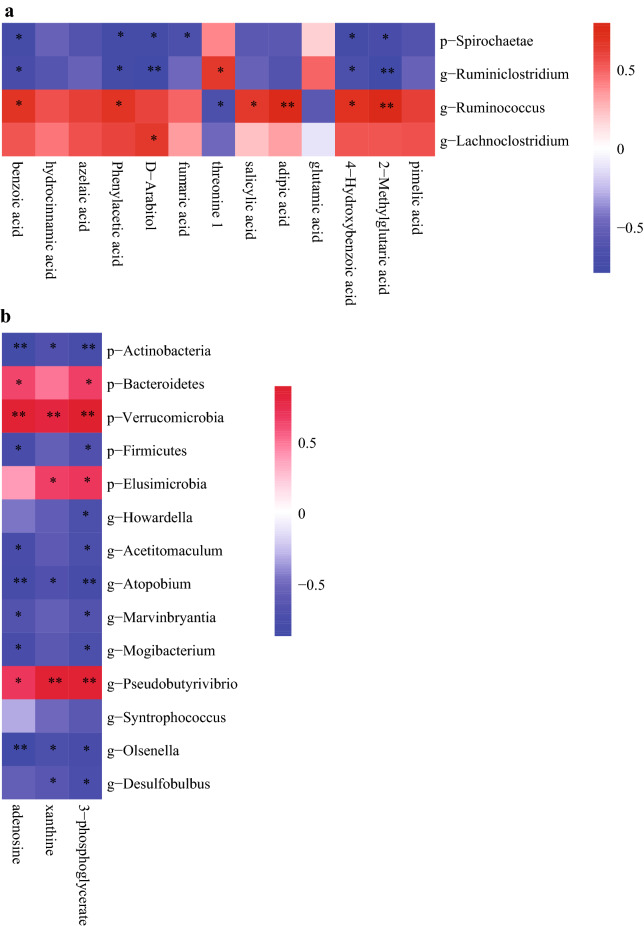
Correlation between difference bacteria and difference metabolite in the rumen fluid of fermented rice straw group and fresh corn Stover group at 30 (**a**) and 90 (**b**) days of feeding. The color was according to the Spearman correlation coefficient distribution. Asterisks indicate significant difference (*P* < 0.05).

Metabolites and bacteria significantly differing between treatment groups at d 30 and d 90 were used in a Spearman correlation analysis. At d 30 (Fig. 6a), the relative abundances of p-Spirochaetae and g-*Ruminiclostridium* were negatively correlated with levels of 2-methylglutaric acid (*P* = 0.029 and *P* = 0.006), 4-hydroxybenzoic acid (*P* = 0.013 and *P* = 0.048), *D*-arabitol (*P* = 0.011 and *P* = 0.008), phenylacetic acid (*P* = 0.016 and *P* = 0.013), and benzoic acid (*P* = 0.011 and *P* = 0.029), respectively. The relative abundance of p-Spirochaetae was negatively correlated with fumaric acid level (*P* = 0.038). The relative abundance of g-*Ruminiclostridium* was positively correlated with threonine level (*P* = 0.048). The relative abundance of g-*Ruminococcus* was positively correlated with levels of 2-methylglutaric acid (*P* = 0.008), 4-hydroxybenzoic acid (*P* = 0.025), adipic acid (*P* = 0.006), salicylic acid (*P* = 0.043), phenylacetic acid (*P* = 0.033) and benzoic acid (*P* = 0.033), respectively. At d 90 (Fig. 6b), the relative abundances of p-Actinobacteria, g-*Atopobium*, and g-*Olsenella* were negatively correlated with levels of 3-phosphoglycerate (*P* = 0.009, *P* = 0.008 and *P* = 0.013), xanthine (*P* = 0.029, *P* = 0.029 and *P* = 0.022) and adenosine (*P* = 0.002, *P* = 0.006 and *P* = 0.001), respectively. The relative abundances of p-Verrucomicrobia and g-*Pseudobutyrivibrio* were positively correlated with levels of 3-phosphoglycerate (*P* = 0.001 and *P* = 0.002), xanthine (*P* = 0.006 and *P* = 0.002) and adenosine (*P* = 0.002 and *P* = 0.029), respectively. The relative abundance of p-Bacteroidetes was positively correlated with levels of 3-phosphoglycerate (*P* = 0.043) and adenosine (*P* = 0.048), respectively. The relative abundance of p-Firmicutes was negatively correlated with levels of 3-phosphoglycerate (*P* = 0.029) and adenosine (*P* = 0.013), respectively.

## Discussion

The rumen is a complex microecosystem that processes complex dietary carbohydrates into VFAs such as acetate, propionate, and butate^19^, which are the main energy sources for ruminants^20^. Data indicated that fermentation efficiency may be improved by increasing VFA and decreasing methane (CH_4_) production^21^ and increasing rumen VFA levels is a concern when ruminants are fed basal diets comprising cereal residues^22^. Rumen VFA production is contingent upon the chemical properties of cereal residues and the microbial communities in the rumen^23^ and treatment of cereal residues with various additives may degrade the lignocellulose complexes into simpler, readily fermentable materials^24^. Many studies demonstrated that treatment of wheat straw with enzymes enhanced neutral detergent fiber (NDF) digestion and fermentation^25^, addition of probiotics and fibrolytic enzymes to paddy straw diet significantly improved nutrient degradability and total rumen VFA^26^ and use of fibrolytic enzymes improved the ruminal fermentation characteristics of rice straw^27^ and increased the apparent digestibility of neutral and acid detergent fiber of rice straw based total mixed ration^28^. It is reported that the cuticle wax silica layer and lignin impede microbial and enzymatic cellulose and hemicellulose degradation^10^ and the cuticle wax silica layer must be removed before enzymatic digestion and microbial attachment can proceed^29^. Data showed that the cuticle wax silica layer on straw may be removed by fermentation with *Streptomyces griseorubens* C-5^30^ and lignocellulose can be hydrolyzed by acetic acid treatment^31^. Rice straw has higher lignin content and thicker cuticle wax silica layers than fresh corn Stover^12^, this results in a lower palatability and digestibility compared rice straw to corn Stover^32^, some studies reported that ruminants had higher dry matter intake and nutrients digestibility when fed treated rice straw compared to untreated rice straw^33,34^. Data in this study indicated that relative to beef cattle fed a diet based on untreated fresh corn Stover, cattle fed a diet based on fermented rice straw with probiotics and enzymes had numerically higher dry matter intake (*P* = 0.433) and significantly higher levels of acetic acid and propionate in rumen liquid at d 60 (*P* = 0.022, *P* = 0.043) and d 90 (*P* = 0.023, *P* = 0.014), respectively. This increased levels of acetic acid and propionate should be related to the increase in dry matter intake and fermentable sugars of fermented rice straw, some experiments found that rice straw can release more fermentable surgars after a biotreatment^35^, solid-state fermentation of fibrous materials by *Bacillus subtilis* can lower cellulose, hemicellulose and lignin levels and raise arabinose, xylose and glucose content^36^, Bacilli can also synthesize pectin lyase to break down pectin^37^.

Dietary composition influences both rumen VFA level and microbial community structure^38–40^. Previous studies showed that Firmicutes and Bacteroidetes predominated at the phylum level and there were relatively more Bacteroidetes than Firmicutes in the rumen fluid of animals fed high-forage or high-concentrate diets^41–46^. These observations are consistent with our findings. The present study indicated that Firmicutes and Bacteroidetes comprised > 90% of all bacterial populations in rumen fluid. Cattle fed fermented rice straw had higher proportions of Bacteroidetes and lower proportions of Firmicutes in their rumen fluid than those fed fresh corn Stover. One possible explanation is that cattle fed fermented rice straw has a higher dry matter intake than those fed fresh corn Stover, because the proportions of Bacteroidetes in buffalo rumen fluid increased with the increased fibous materials intake^47^. Firmicutes participate in carbohydrate digestion and fiber component (cellulose and hemicellulose) utilization^41,48^. However, it is mainly the Bacteroidetes that degrade complex polysaccharides (cellulose, hemicellulose, pectin, and xylan) as they bear more genes encoding glycoside hydrolases and polysaccharide lyases than the Firmicutes and other bacterial phyla^49–51^. Bacteroidetes also degrade oligosaccharides^52^ and proteins^48^ by secreting carbohydrate-digesting enzymes^50^ and dipeptidyl peptidases^53^. Comparatively more xylan was released by the hemicellulose breakdown during rice straw fermentation and it accelerated Bacteroidetes growth^54^. For this reason, cattle fed fermented rice straw had higher Bacteroidetes levels in their rumen fluid than those fed fresh corn Stover. Bacteroidetes ferment complex carbohydrates into acetic acid and propionate^55^. Thus, there was greater acetic acid and propionate production in the rumen fluid of fermented rice straw-fed cattle than in that of fresh corn Stover-fed cattle as the former had relatively higher numbers of Bacteroidetes and Fibrobacteres. With increasing Bacteroidetes relative abundances, the use of H_2_ increases, resulting propionate levels increase and methane levels decrease^2^. Maximizing the flow of metabolic hydrogen away from methane and towards propionate might increase the efficiency of feed energy conversion in the rumen^56^. The rumen fluid of fermented rice straw-fed cattle had more Bacteroidetes than that of fresh corn Stover-fed cattle, thus, fermented rice straw may more effectively improve the efficiency of feed energy conversion in the rumen than fresh corn Stover. Moreover, cattle fed fermented rice straw had higher proportions of Fibrobacteres (*P* = 0.035) and lower proportions of Spirochaetae (*P* = 0.018) and Actinobacteria (*P* = 0.033 and *P* = 0.001) than those fed fresh corn Stover. The Fibrobacteres comprise highly efficient cellulolytic bacteria that break down cellulose, xylan, and cellobiose and ferment their degradation products to acetic acid^57,58^. The proportions of short chain fatty acids (SCFAs) produced increase with the number of Fibrobacteres^59^. Spirochaetae and Actinobacteria produce several enzymes that break down plant biomass into simple sugars or fatty acids^60,61^. Nevertheless, these phyla had only minor impacts on the total VFA level in the rumen fluid as their total abundance for both treatment groups was < 1.6%.

Here, *Prevotella* were the dominant bacteria in rumen fluid. However, there was no significant difference in *Prevotella* abundance between treatments (*P* = 0.293, *P* = 0.386 and *P* = 0.783). Cattle fed fermented rice straw with lower fiber (NDF and ADF) content had higher relative abundances of *Prevotella* than those fed fresh corn Stover with higher fiber (NDF and ADF) content. These findings aligned with an earlier report stating that high concentrate levels in the ruminant diet promoted *Prevotella* growth^62^. In contrast, goats fed high-fiber diets had higher relative *Prevotella* abundance in their rumens than those fed low-fiber diets^63^. *Prevotella* secrete hemicellulolytic and proteolytic enzymes^64^ and degrade polysaccharides such as xylan, pectin, and starch^65^. *Prevotella* activity is positively associated with butyrate level^13^. Therefore, cattle fed fermented rice straw had higher butyric acid levels in their rumen fluid than those fed fresh corn Stover at d 60 (*P* = 0.108) and d 90 (*P* = 0.255), respectively.

Cattle fed fermented rice straw also had significantly higher numbers of *Ruminococcus* (*P* = 0.023)*, Saccharofermentans* (*P* = 0.036)*, Treponema* (*P* = 0.043)*, Lachnoclostridium* (*P* = 0.020)*, Ruminobacter* (*P* = 0.043), and *Pseudobutyrivibrio* (*P* = 0.008) in their rumen fluid than those fed fresh corn Stover. *Ruminococcus, Saccharofermentans,* and *Pseudobutyrivibrio* are anaerobic cellulolytic bacteria that ferment cellulose, hemicelluloses, and other polysaccharides into acetate and succinate^66–69^. Certain *Ruminococcus* and *Saccharofermentans* species can shift from succinate to acetate and propionate^67,69^, *Ruminococcus* and *Prevotella* secrete enzymes that convert fumaric acid into succinic acid^70,71^. Here, it was determined that the *Ruminococcus* level was positively correlated with the fumaric acid content. Fumaric acid inhibits rumen methane production^72^. Cattle fed fermented rice straw had higher fumaric acid levels in their rumen than those fed fresh corn Stover. Thus, fermented rice straw may more effectively lower methane generation and improve feed energy conversion efficiency than fresh corn Stover. Methane production decreases with pH and is completely inhibited at pH < 6.0^73^. In the present study, the rumen fluid of the fermented rice straw-fed cattle had a lower pH than that of the fresh corn Stover-fed cattle. *Pseudobutyrivibrio* degrade hemicellulose and ferment carbohydrates to butyric acid^74^. The proportion of *Pseudobutyrivibrio* was higher in the rumen fluid of the fermented rice straw-fed cattle than in that of the fresh corn Stover-fed cattle. For this and other reasons, the butyric acid content was higher in the rumen fluid of fermented rice straw-fed cattle than in that of fresh corn Stover-fed cattle.

*Treponema* degrade pectin, xylan, and fructan and hydrolyze cellobiose, xylose, arabinose, and galacturonic acid^75–77^. Pectin fermentation by pectinolytic bacteria such as *Treponema* yields acetate^78^. *Treponema* and *Prevotella* grow faster on high-pectin than low-pectin media^77,79^. The pectin content in rice straw is higher than that in corn Stover. Moreover, fermentation releases free pectin from the carbohydrate polymer. Thus, there was a higher level of free pectin in fermented rice straw than fresh corn Stover. Consequently, cattle fed fermented rice straw had higher numbers of *Treponema* and *Prevotella* in their rumen fluid than cattle fed fresh corn Stover. *Lachnoclostridium* are anaerobic bacteria that degrade cellulose and related plant cell wall polysaccharides to simple sugars that can be used as substrates for microbial growth and fermentation^80^. *Ruminobacter* are amylolytic bacteria and their growth is promoted in the rumens of cattle fed diets high in starch and fermentable sugars and low in fiber^81^. To avoid lowering the pH below 5.5, *Ruminobacter* ferment carbohydrates into formate, acetate, propionate, and succinate instead of lactic acid^82,83^. Rice straw fermented with *Bacillus subtilis, Enterococcus faecalis*, cellulase, and xylanase generated higher levels of fermentable sugars than fresh corn Stover. Thus, fermented rice straw more effectively enhanced *Ruminobacter* growth and maintained rumen pH balance than fresh corn Stover.

Studies demonstrated that phenylacetic acid can enhance cellulose degradation and growth of several strains of *Ruminococcus albus*^84,85^, fumaric acid can increase the production of propionate^86,87^ and benzoic acid not only can increase the production of propionate and butyric acid but also can improve the digestibility of energy and neutral detergent fibre^88^. Data in this experiment showed that beef cattle fed a diet based on fermented rice straw had significantly higher levels of phenylacetic acid, hydrocinnamic acid, azelaic acid, D-arabitol, fumaric acid, salicylic acid, benzoic acid, adipic acid, 4-hydroxybenzoic acid, 2-methylglutaric acid, pimelic acid, adenosine, xanthine and 3-phosphoglycerate compared beef cattle fed a diet based on fresh corn Stover. The increased levels of phenylacetic acid, fumaric acid and benzoic acid didn’t significantly increase the propionate production (*P* = 0.809) but the increased levels of phenylacetic acid and benzoic acid significantly elevated the relative abundance of g-*Ruminococcus* (*P* = 0.033 and *P* = 0.033) at d 30 in the rumen liquid of beef cattle fed fermented rice straw based diet compared with beef cattle fed fresh corn Stover based diet.

## Conclusions

Feeding rice straw co-fermented with probiotics and enzymes, instead of fresh corn Stover to beef cattle altered the ruminal bacterial community toward increased relative abundance of p-Fibrobacteres, p-Bacteroidetes, g-*Ruminococcus*, g-*Lachnoclostridium*, g-*Pseudobutyrivibrio*, g-*Saccharofermentans*, g-*Treponema* and g-*Ruminobacter*, elevated the rumen metabolites toward *Ruminococcus* growth and propionate production, changed the rumen fermentation pattern from acetate to propionate.

## Methods

### Preparation of fermented rice straw and fresh corn Stover

A starter culture was prepared and it consisted of *Bacillus subtilis* (viable cell count 1.0 × 10^11^ CFU g^-1^, Jiangxi Xinwei Biotechnology Co., Ltd, China), *Enterococcus faecalis* (viable cell count 1.0 × 10^11^ CFU g^-1^, Jiangxi Xinwei Biotechnology Co., Ltd, China), cellulase (activity 1.0 × 10^4^ U g^-1^, Shandong Xindeli biology Co., Ltd, China), and xylanase (activity 5.0 × 10^4^ U g^-1^, Shandong Xindeli biology Co., Ltd, China) at a 1:1:10:30 ratio. Then 6.2 g starter culture, 75 g brown sugar, and 15 g sodium chloride (NaCl) were dissolved in 1.3 kg water to ferment 1 kg rice straw at ~ 13% moisture content. Rice straw was chopped into segments 3–5 cm long, placed in polypropylene bags^89^, and weighed, then the fermentation fluid was sprayed uniformly on the rice straw at a 1:1.3 rice straw:fluid ratio. The air was evacuated from the bags and they were sealed with plastic cable ties. The sealed bags were stored for 14 d before their contents were fed to the cattle. Fresh corn Stover was chopped into segments 3–5 cm long then fed as is to the cattle.

### Animals and experimental design

All experimental procedures involving animal care and sampling were approved by the Ethics Committee for Animal Experimentation of Jiangxi Agricultural University and all methods were performed in accordance with the relevant guidelines and regulations. Twenty Simmental hybrid bulls at 13–14 months of age were randomly divided into two groups based on the initial average body weight (369.60 ± 18.53 kg vs. 371.50 ± 15.84 kg, *P* = 0.939) and fed ad libitum, each group had ten bulls. One group was fed rice straw co-fermented with probiotics and enzymes as roughage while the other group received fresh corn Stover as roughage. The trial ran for 90 days. Composition and nutrient levels of the basal diet are as follows (Table 6).

**Table.6:** Composition and nutrient levels of the basal diet (air-dry basis) %

	Fermented rice straw group (Group A)	Fresh corn Stover group (Group B)
**Ingredients**		
Corn	16	16
Soybean	2.8	2.8
Premix^1^	0.8	0.8
NaHCO_3_	0.2	0.2
Nacl	0.2	0.2
Brewer's grains	40	40
Fermented rice straw	40	0
Fresh corn Stover	0	40
**Nutrient levels**^2^		
DM	42.23	37.79
NEmf (MJ/kg)	4.92	5.18
CP	10.03	11.25
NDF	33.18	34.81
ADF	15.67	17.43

^1^Per kg of premix included the following: VitA 200 000 IU, VitD3 25 000 IU, VitE 4 000 IU, Fe 3,500 mg, Mn 2000 mg, Zn 1,500 mg, Cu 550 mg, I 30 mg, Se 15 mg, Co 15 mg, Ca 150 g, P 60 g. ^2^ NEmf were calculated values, while others were measured values.

### Sample collection

On the day of 30, 60 and 90 in experimental period, about 100 mL rumen fluid was collected at 8:00 am with an esophageal tube vacuum pump sampling device (Anscitech, Wuhan Kelibo Equipment co. Ltd., Wuhan, China) from each animal and delivered into a 500-mL beaker. The rumen fluid was separated into solid and liquid fractions by pressing it through four layers of muslin cloth. The pH of rumen liquid was immediately measured, 8-mL liquid aliquots were placed in seven 10-mL sterile centrifuge tubes and all tubes with rumen liquid samples were immediately frozen in liquid nitrogen and stored at − 80 °C until subsequent 16S rRNA sequencing and metabolomics analysis.

### Chemical analysis

Rumen liquid portion pH was measured with a digital pH meter (Leici PHB-4; Shanghai INESA Scientific Instrument Co. Ltd., Shanghai, China) precalibrated with standard pH buffers. VFAs were analyzed by gas chromatography (GC-2014; Shimadzu Corp., Kyoto, Japan) according to a previously published method^90^. Lactic acid concentration was determined with an assay kit according to the manufacturer's instructions (Nanjing Jiancheng Bioengineering Institute, Nanjing, China).

### Sequencing of rumen microbiota

Genomic DNA was isolated from rumen liquid portion according to a previously published method^91^. PCR amplification was performed with the universal primers 515F and 926R and targeted the V4–V5 regions of bacterial 16S rRNA^92^. The PCR products were collected with an AxyPrepDNA gel recovery kit (Aisijin Biotechnology Co. Ltd., Hangzhou, China) and sequenced on Miseq 2 × 300-bp platform (Illumina, San Diego, CA, USA).

### GC–TOF/MS analysis

Metabolites in samples of the rumen liquid portion from the animals in both treatment groups were compared by GC–TOF/MS according to a previously published method^93^. Brief procedures are as follows: Firstly, sample was mixed with methanol-chloroform and ribitol in EP tube by vortexing and extracted, then EP tube was centrifuged and supernatant was removed from EP tube and pipetted into a 2 mL glass vial. Secondly, glass vial with supernatant was dried and incubated with methoxymethyl amine salt, another incubation followed with Bis-(trimethylsilyl)-trifluoroacetamide, then the glass vial was mixed with fatty acid methyl esters for GC–TOF–MS analysis. The GC–TOF–MS analysis was performed using an Agilent 7,890 gas chromatograph system coupled with a Pegasus HT time-of-flight mass spectrometer according to the manufacture’s instruction.

### Statistical analysis

#### Rumen fluid analysis

Differences in the mean pH, VFA, and lactic acid concentrations between groups were analyzed by *t*-test in SPSS v. 17.0 (IBM Corp., Armonk, NY, USA). Data are means ± SEM. *P* < 0.05 was considered statistically significant.

#### 16S rRNA sequence analysis

Data were filtering according to a previously published method^94^. High-quality sequences were subjected to OTU (operational taxonomic unit) analysis in Usearch v. 7.1^95^. OTUs with > 97% similarity were clustered^96^. The OTU clusters were compared against the Silva 128 database to identify the microbial classifications of the representative OTU sequences. Significant differences between treatment groups in terms of their microorganism profiles were identified by *t*-tests. *P* < 0.05 was considered statistically significant. Based on the precalculated Silva 128 database, Tax4FUN v. 0.3.1 (https://tax4fun.gobics.de/)^97^ was run on the abundance predictions of the Kyoto Encyclopedia of Genes and Genomes (KEGG) orthologs and bacterial pathways^98,99^. A two-sided Welch’s *t*-test was used for two-group bacterial functional prediction analyses. Functional differences between treatment groups were compared by Statistical Analysis of Metagenomic Profiles (STAMP)^100^.

#### GC–MS data acquisition and analysis

Chroma TOF v. 4.3X software (https://www.lecosoftware.com/chromatof) and the LECO-Fiehn Rtx5 database (LECO Corporation, St. Joseph, MI, USA) were used to identify raw peaks, filter and calibrate data baselines, align and identify the peaks, integrate their areas, and perform a deconvolution analysis^101^. Mass spectrum and retention index matching were considered in metabolite identification. Peaks detected in < 50% and RSD in > 30% of the QC samples were removed^102^. Differential metabolite screening was conducted using previously published methods^93^ and peaks with similarity greater than 700, variable importance projection (VIP) exceeding 1.0 and *P* < 0.05 by *t*-test were selected as the reliable differentially expressed metabolites. KEGG database was used to search for differential metabolite pathways^103,104^.

#### Correlation analyses of microbiomes and metabolomes

The cor.test function in R v. 3.5.1 was used to calculate the Spearman correlation coefficients among differential bacteria and differential metabolites in the rumen fluid of the treatment groups at d 30 and d 90, statistical significance was set at *P* < 0.05. The pheatmap package v. 1.0.12 (https://CRAN.R-project.org/package=pheatmap) was used to plot the correlation heatmap among the different bacteria and metabolites.
